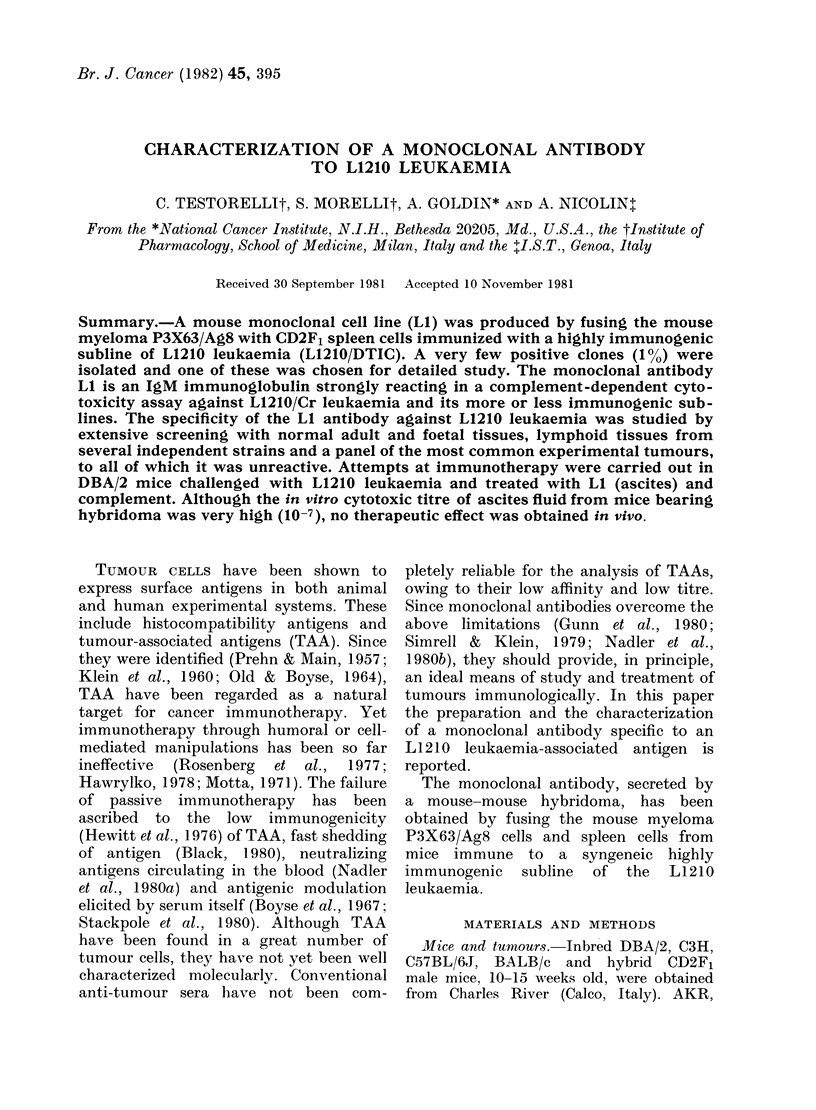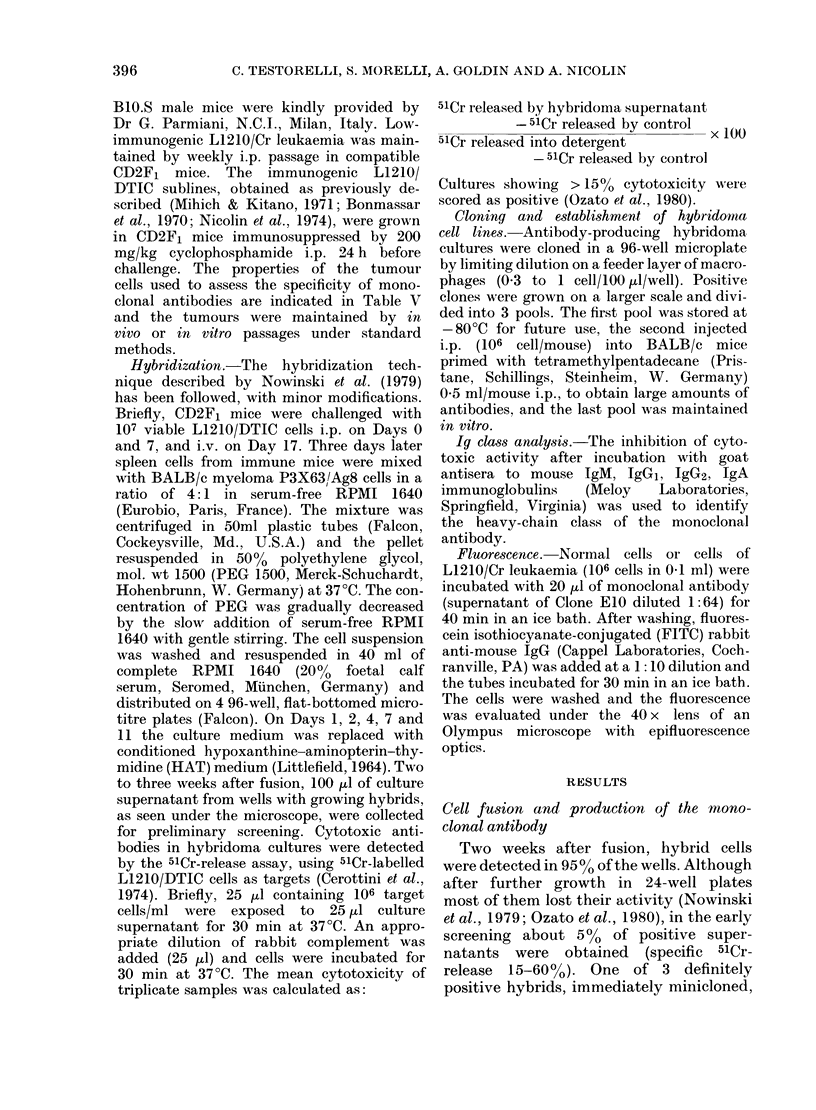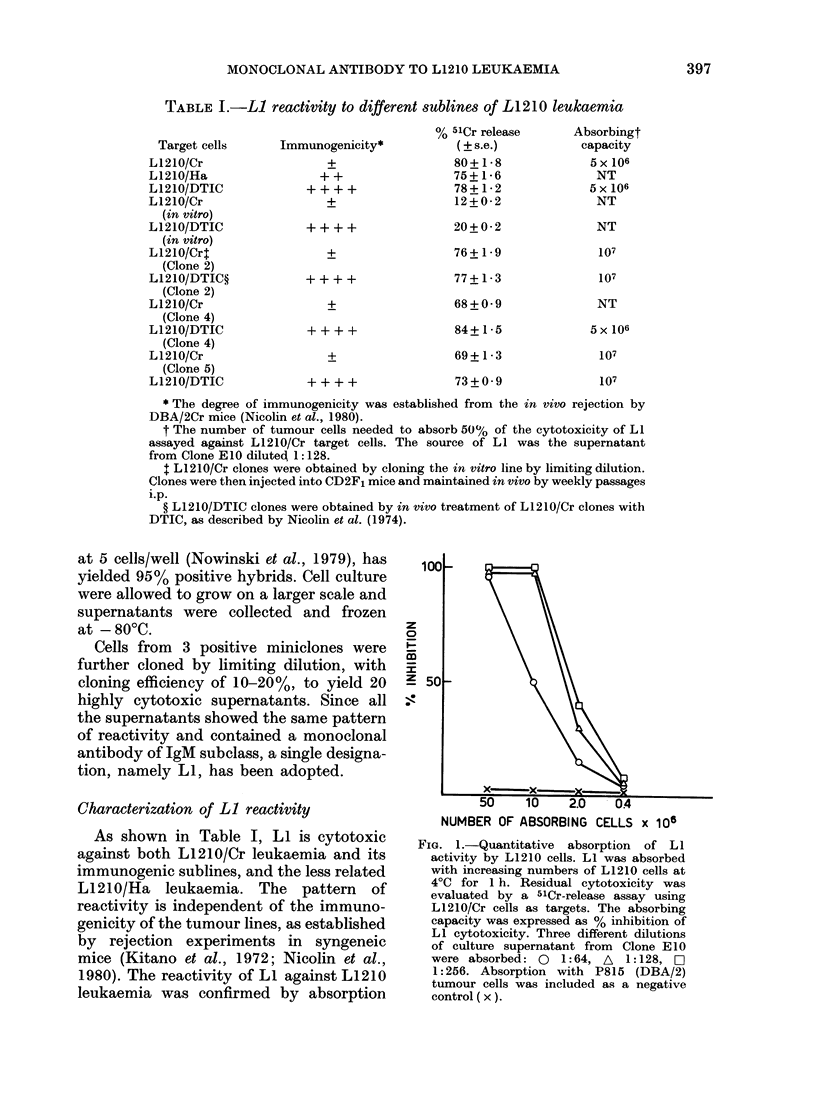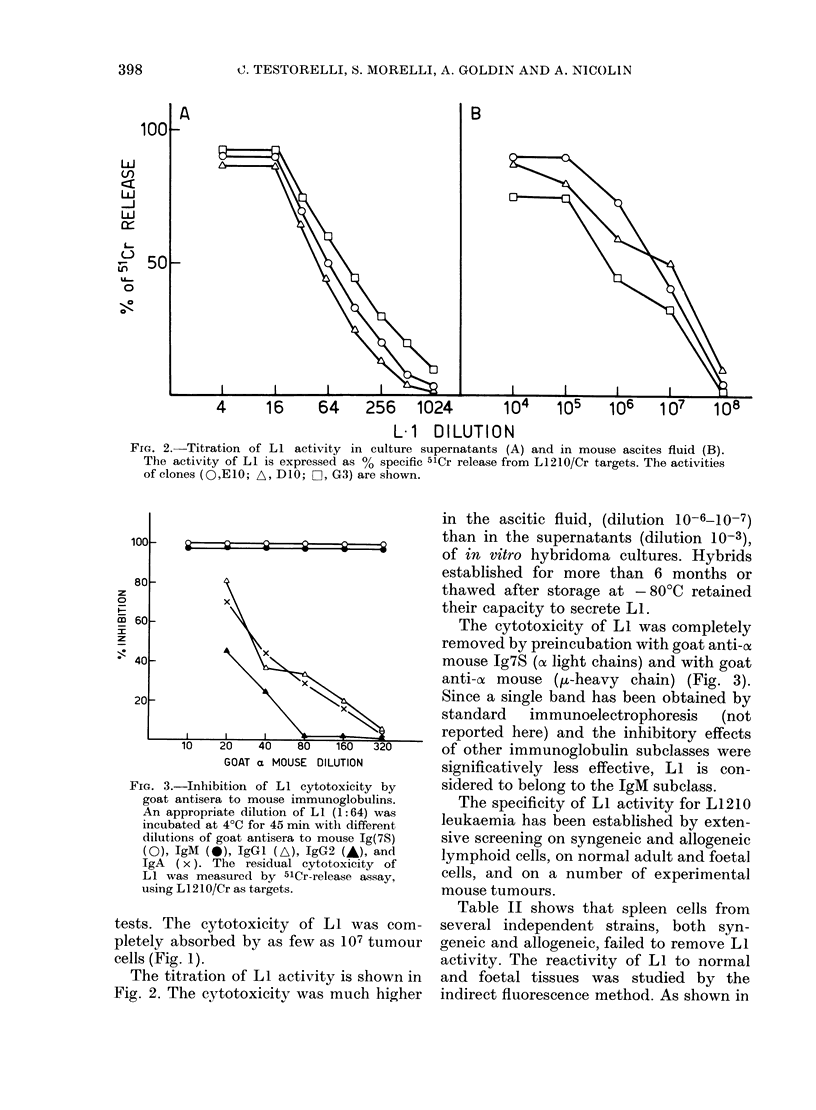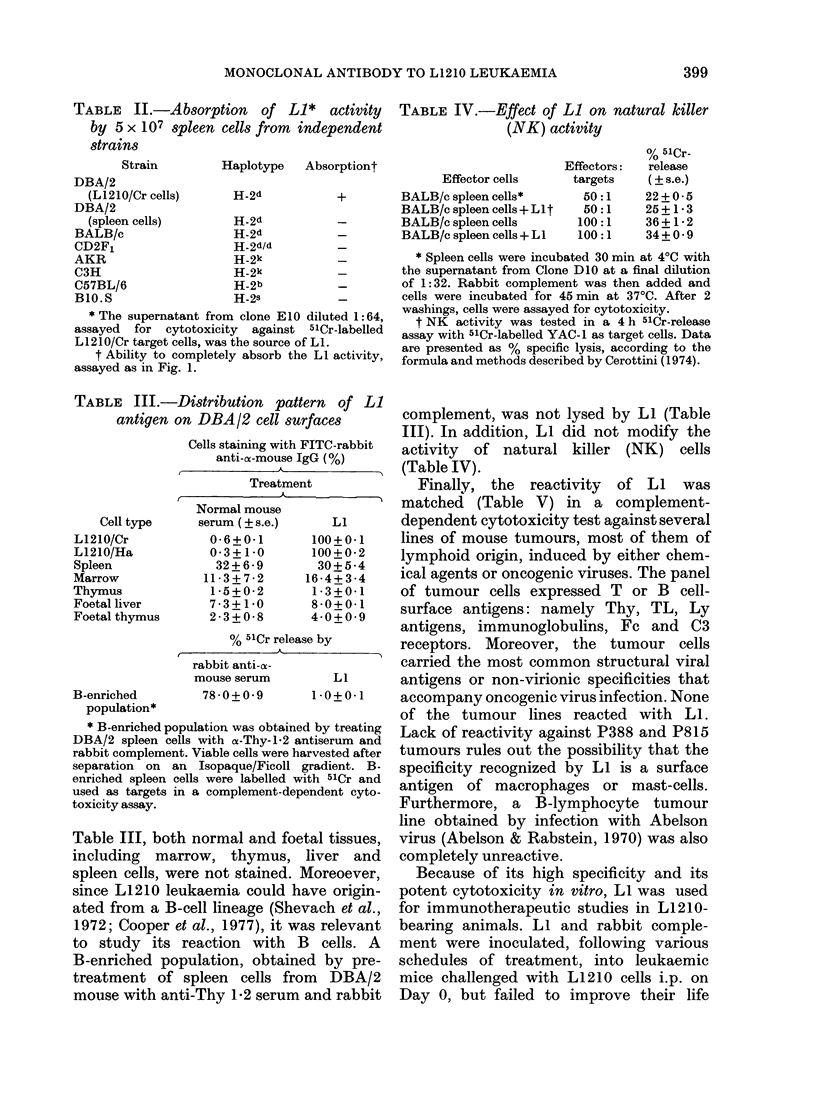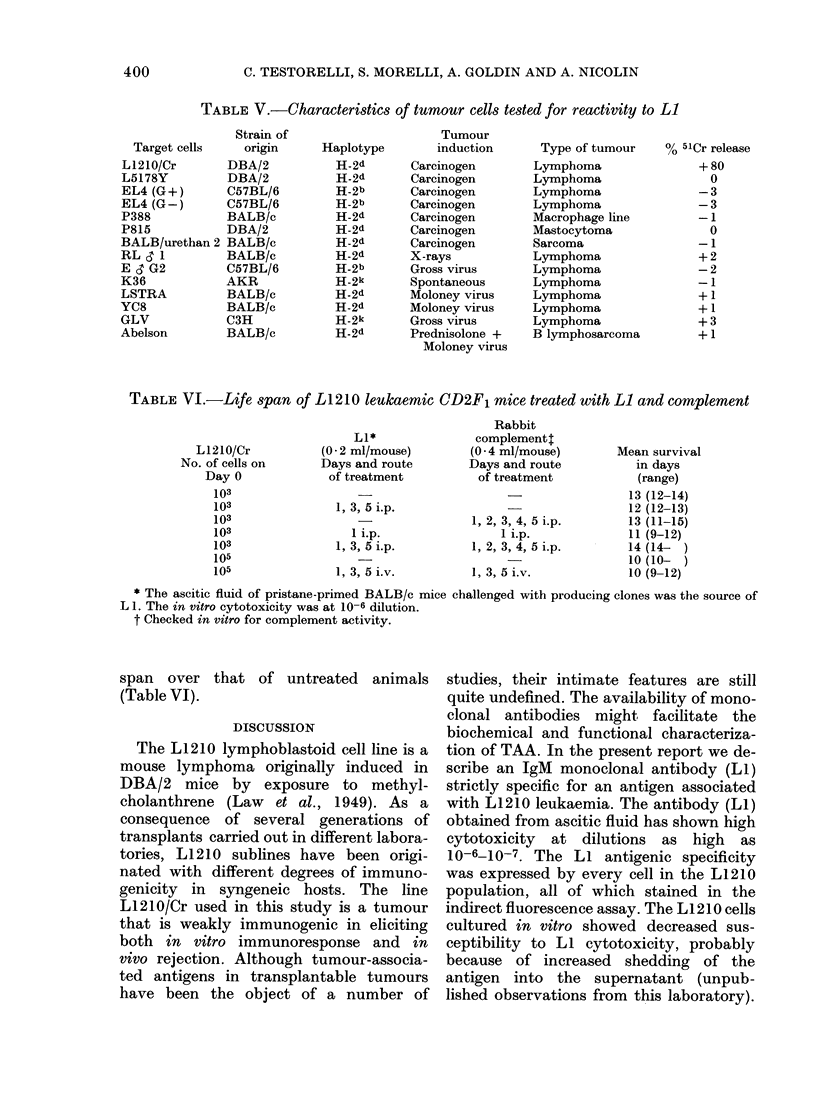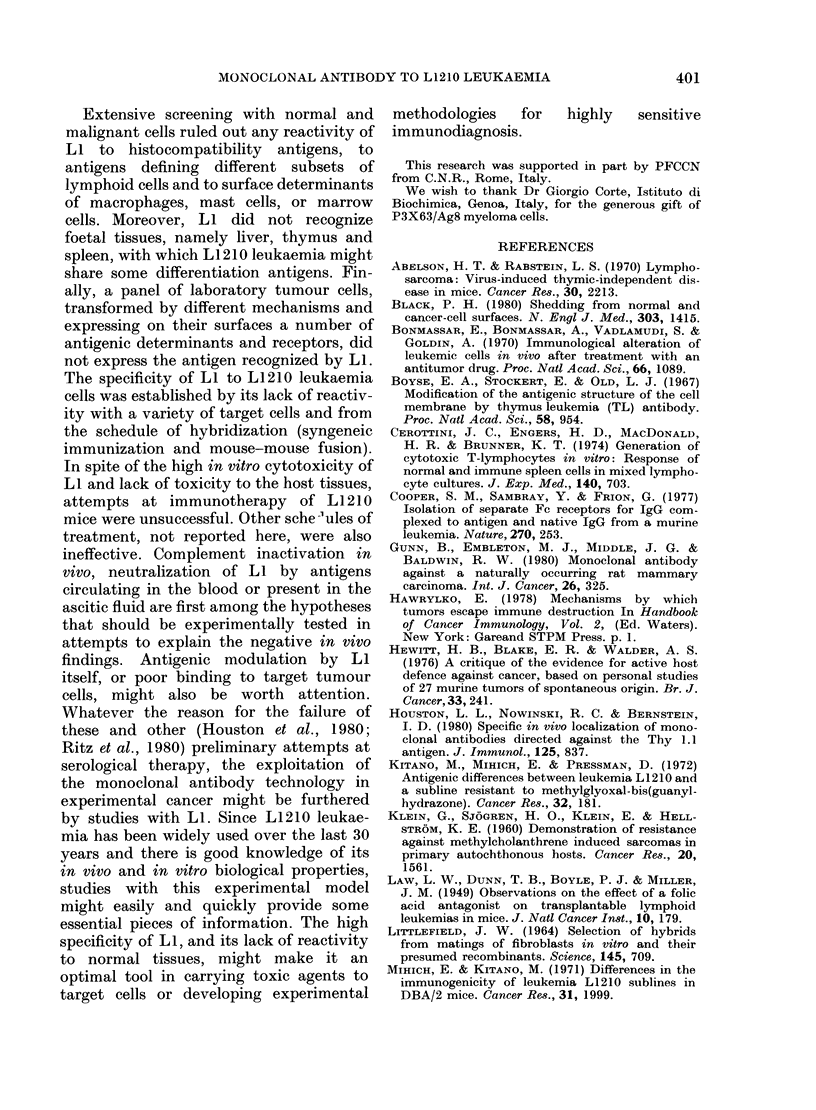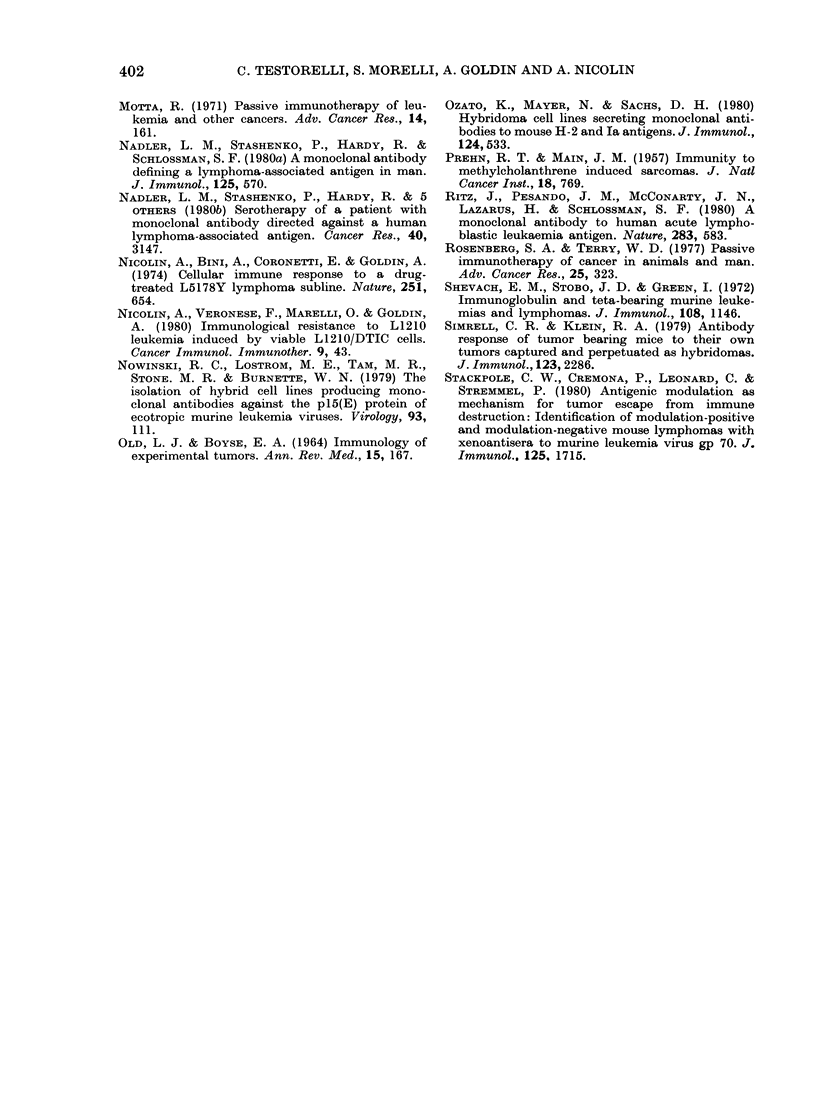# Characterization of a monoclonal antibody to L1210 leukaemia.

**DOI:** 10.1038/bjc.1982.67

**Published:** 1982-03

**Authors:** C. Testorelli, S. Morelli, A. Goldin, A. Nicolin

## Abstract

A mouse of monoclonal cell line (L1) was produced by fusing the mouse myeloma P3X63/Ag8 with CD2F1 spleen cells immunized with a highly immunogenic subline of L1210 leukaemia (L1210/DTIC). A very few positive clones (1%) were isolated and one of these was chosen for detailed study. The monoclonal antibody L1 is an IgM immunoglobulin strongly reacting in a complement-dependent cytotoxicity assay against L1210/Cr leukaemia and its more or less immunogenic sublines. The specificity of the L1 antibody against L1210 leukaemia was studied by extensive screening with normal adult and foetal tissues, lymphoid tissues from several independent strains and a panel of the most common experimental tumours, to all of which it was unreactive. Attempts at immunotherapy were carried out in DBA/2 mice challenged with L1210 leukaemia and treated with L1 (ascites) and complement. Although the in vitro cytotoxic titre of ascites fluid from mice bearing hybridoma was very high (10(-7)), no therapeutic effect was obtained in vivo.


*Br. J. Cancer* (1982) **45**, 395

### CHARACTERIZATION OF A MONOCLONAL ANTIBODY TO L1210 LEUKAEMIA

C. TESTORELLI†, S. MORELLI†, A. GOLDIN* AND A. NICOLIN‡

*From the *National Cancer Institute, N.I.H., Bethesda* 20205, *Md., U.S.A., the †Institute of Pharmacology, School of Medicine, Milan, Italy and the ‡I.S.T., Genoa, Italy*

Received 30 September 1981 Accepted 10 November 1981

**Summary.—A mouse monoclonal cell line (L1) was produced by fusing the mouse myeloma P3X63/Ag8 with $CD2F_1$ spleen cells immunized with a highly immunogenic subline of L1210 leukaemia (L1210/DTIC). A very few positive clones (1%) were isolated and one of these was chosen for detailed study. The monoclonal antibody L1 is an IgM immunoglobulin strongly reacting in a complement-dependent cytotoxicity assay against L1210/Cr leukaemia and its more or less immunogenic sublines. The specificity of the L1 antibody against L1210 leukaemia was studied by extensive screening with normal adult and foetal tissues, lymphoid tissues from several independent strains and a panel of the most common experimental tumours, to all of which it was unreactive. Attempts at immunotherapy were carried out in DBA/2 mice challenged with L1210 leukaemia and treated with L1 (ascites) and complement. Although the *in vitro* cytotoxic titre of ascites fluid from mice bearing hybridoma was very high ($10^{-7}$), no therapeutic effect was obtained *in vivo*.**

TUMOUR CELLS have been shown to express surface antigens in both animal and human experimental systems. These include histocompatibility antigens and tumour-associated antigens (TAA). Since they were identified (Prehn & Main, 1957; Klein *et al.*, 1960; Old & Boyse, 1964), TAA have been regarded as a natural target for cancer immunotherapy. Yet immunotherapy through humoral or cell-mediated manipulations has been so far ineffective (Rosenberg *et al.*, 1977; Hawrylko, 1978; Motta, 1971). The failure of passive immunotherapy has been ascribed to the low immunogenicity (Hewitt *et al.*, 1976) of TAA, fast shedding of antigen (Black, 1980), neutralizing antigens circulating in the blood (Nadler *et al.*, 1980*a*) and antigenic modulation elicited by serum itself (Boyse *et al.*, 1967; Stackpole *et al.*, 1980). Although TAA have been found in a great number of tumour cells, they have not yet been well characterized molecularly. Conventional anti-tumour sera have not been completely reliable for the analysis of TAAs, owing to their low affinity and low titre. Since monoclonal antibodies overcome the above limitations (Gunn *et al.*, 1980; Simrell & Klein, 1979; Nadler *et al.*, 1980*b*), they should provide, in principle, an ideal means of study and treatment of tumours immunologically. In this paper the preparation and the characterization of a monoclonal antibody specific to an L1210 leukaemia-associated antigen is reported.

The monoclonal antibody, secreted by a mouse–mouse hybridoma, has been obtained by fusing the mouse myeloma P3X63/Ag8 cells and spleen cells from mice immune to a syngeneic highly immunogenic subline of the L1210 leukaemia.

### MATERIALS AND METHODS

*Mice and tumours.*—Inbred DBA/2, C3H, C57BL/6J, BALB/c and hybrid $CD2F_1$ male mice, 10–15 weeks old, were obtained from Charles River (Calco, Italy). AKR,

B10.S male mice were kindly provided by Dr G. Parmiani, N.C.I., Milan, Italy. Low-immunogenic L1210/Cr leukaemia was maintained by weekly i.p. passage in compatible $CD2F_1$ mice. The immunogenic L1210/DTIC sublines, obtained as previously described (Mihich & Kitano, 1971; Bonmassar *et al.*, 1970; Nicolin *et al.*, 1974), were grown in $CD2F_1$ mice immunosuppressed by 200 mg/kg cyclophosphamide i.p. 24 h before challenge. The properties of the tumour cells used to assess the specificity of monoclonal antibodies are indicated in Table V and the tumours were maintained by *in vivo* or *in vitro* passages under standard methods.

*Hybridization.*—The hybridization technique described by Nowinski *et al.* (1979) has been followed, with minor modifications. Briefly, $CD2F_1$ mice were challenged with $10^7$ viable L1210/DTIC cells i.p. on Days 0 and 7, and i.v. on Day 17. Three days later spleen cells from immune mice were mixed with BALB/c myeloma P3X63/Ag8 cells in a ratio of 4:1 in serum-free RPMI 1640 (Eurobio, Paris, France). The mixture was centrifuged in 50ml plastic tubes (Falcon, Cockeysville, Md., U.S.A.) and the pellet resuspended in 50% polyethylene glycol, mol. wt 1500 (PEG 1500, Merck-Schuchardt, Hohenbrunn, W. Germany) at 37°C. The concentration of PEG was gradually decreased by the slow addition of serum-free RPMI 1640 with gentle stirring. The cell suspension was washed and resuspended in 40 ml of complete RPMI 1640 (20% foetal calf serum, Seromed, München, Germany) and distributed on 4 96-well, flat-bottomed microtitre plates (Falcon). On Days 1, 2, 4, 7 and 11 the culture medium was replaced with conditioned hypoxanthine–aminopterin–thymidine (HAT) medium (Littlefield, 1964). Two to three weeks after fusion, 100 $\mu$l of culture supernatant from wells with growing hybrids, as seen under the microscope, were collected for preliminary screening. Cytotoxic antibodies in hybridoma cultures were detected by the $^{51}$Cr-release assay, using $^{51}$Cr-labelled L1210/DTIC cells as targets (Cerottini *et al.*, 1974). Briefly, 25 $\mu$l containing $10^6$ target cells/ml were exposed to 25 $\mu$l culture supernatant for 30 min at 37°C. An appropriate dilution of rabbit complement was added (25 $\mu$l) and cells were incubated for 30 min at 37°C. The mean cytotoxicity of triplicate samples was calculated as:

$$\frac{^{51}\text{Cr released by hybridoma supernatant} - {}^{51}\text{Cr released by control}}{^{51}\text{Cr released into detergent} - {}^{51}\text{Cr released by control}} \times 100$$

Cultures showing >15% cytotoxicity were scored as positive (Ozato *et al.*, 1980).

*Cloning and establishment of hybridoma cell lines.*—Antibody-producing hybridoma cultures were cloned in a 96-well microplate by limiting dilution on a feeder layer of macrophages (0·3 to 1 cell/100 $\mu$l/well). Positive clones were grown on a larger scale and divided into 3 pools. The first pool was stored at −80°C for future use, the second injected i.p. ($10^6$ cell/mouse) into BALB/c mice primed with tetramethylpentadecane (Pristane, Schillings, Steinheim, W. Germany) 0·5 ml/mouse i.p., to obtain large amounts of antibodies, and the last pool was maintained *in vitro*.

*Ig class analysis.*—The inhibition of cytotoxic activity after incubation with goat antisera to mouse IgM, $IgG_1$, $IgG_2$, IgA immunoglobulins (Meloy Laboratories, Springfield, Virginia) was used to identify the heavy-chain class of the monoclonal antibody.

*Fluorescence.*—Normal cells or cells of L1210/Cr leukaemia ($10^6$ cells in 0·1 ml) were incubated with 20 $\mu$l of monoclonal antibody (supernatant of Clone E10 diluted 1:64) for 40 min in an ice bath. After washing, fluorescein isothiocyanate-conjugated (FITC) rabbit anti-mouse IgG (Cappel Laboratories, Cochranville, PA) was added at a 1:10 dilution and the tubes incubated for 30 min in an ice bath. The cells were washed and the fluorescence was evaluated under the 40× lens of an Olympus microscope with epifluorescence optics.

### RESULTS

### *Cell fusion and production of the monoclonal antibody*

Two weeks after fusion, hybrid cells were detected in 95% of the wells. Although after further growth in 24-well plates most of them lost their activity (Nowinski *et al.*, 1979; Ozato *et al.*, 1980), in the early screening about 5% of positive supernatants were obtained (specific $^{51}$Cr-release 15–60%). One of 3 definitely positive hybrids, immediately minicloned,

TABLE I.—*L1 reactivity to different sublines of L1210 leukaemia*

| Target cells | Immunogenicity* | % $^{51}$Cr release (±s.e.) | Absorbing† capacity |
|---|---|---|---|
| L1210/Cr | ± | 80±1·8 | 5×10$^6$ |
| L1210/Ha | ++ | 75±1·6 | NT |
| L1210/DTIC | ++++ | 78±1·2 | 5×10$^6$ |
| L1210/Cr (*in vitro*) | ± | 12±0·2 | NT |
| L1210/DTIC (*in vitro*) | ++++ | 20±0·2 | NT |
| L1210/Cr‡ (Clone 2) | ± | 76±1·9 | 10$^7$ |
| L1210/DTIC§ (Clone 2) | ++++ | 77±1·3 | 10$^7$ |
| L1210/Cr (Clone 4) | ± | 68±0·9 | NT |
| L1210/DTIC (Clone 4) | ++++ | 84±1·5 | 5×10$^6$ |
| L1210/Cr (Clone 5) | ± | 69±1·3 | 10$^7$ |
| L1210/DTIC | ++++ | 73±0·9 | 10$^7$ |

* The degree of immunogenicity was established from the *in vivo* rejection by DBA/2Cr mice (Nicolin *et al.*, 1980).

† The number of tumour cells needed to absorb 50% of the cytotoxicity of L1 assayed against L1210/Cr target cells. The source of L1 was the supernatant from Clone E10 diluted 1:128.

‡ L1210/Cr clones were obtained by cloning the *in vitro* line by limiting dilution. Clones were then injected into CD2F$_1$ mice and maintained *in vivo* by weekly passages i.p.

§ L1210/DTIC clones were obtained by *in vivo* treatment of L1210/Cr clones with DTIC, as described by Nicolin *et al.* (1974).

at 5 cells/well (Nowinski *et al.*, 1979), has yielded 95% positive hybrids. Cell culture were allowed to grow on a larger scale and supernatants were collected and frozen at −80°C.

Cells from 3 positive miniclones were further cloned by limiting dilution, with cloning efficiency of 10–20%, to yield 20 highly cytotoxic supernatants. Since all the supernatants showed the same pattern of reactivity and contained a monoclonal antibody of IgM subclass, a single designation, namely L1, has been adopted.

### *Characterization of L1 reactivity*

As shown in Table I, L1 is cytotoxic against both L1210/Cr leukaemia and its immunogenic sublines, and the less related L1210/Ha leukaemia. The pattern of reactivity is independent of the immunogenicity of the tumour lines, as established by rejection experiments in syngeneic mice (Kitano *et al.*, 1972; Nicolin *et al.*, 1980). The reactivity of L1 against L1210 leukaemia was confirmed by absorption

FIG. 1.—Quantitative absorption of L1 activity by L1210 cells. L1 was absorbed with increasing numbers of L1210 cells at 4°C for 1 h. Residual cytotoxicity was evaluated by a $^{51}$Cr-release assay using L1210/Cr cells as targets. The absorbing capacity was expressed as % inhibition of L1 cytotoxicity. Three different dilutions of culture supernatant from Clone E10 were absorbed: ○ 1:64, △ 1:128, □ 1:256. Absorption with P815 (DBA/2) tumour cells was included as a negative control (×).

FIG. 2.—Titration of L1 activity in culture supernatants (A) and in mouse ascites fluid (B). The activity of L1 is expressed as % specific $^{51}$Cr release from L1210/Cr targets. The activities of clones (○,E10; △, D10; □, G3) are shown.

FIG. 3.—Inhibition of L1 cytotoxicity by goat antisera to mouse immunoglobulins. An appropriate dilution of L1 (1:64) was incubated at 4°C for 45 min with different dilutions of goat antisera to mouse Ig(7S) (○), IgM (●), IgG1 (△), IgG2 (▲), and IgA (×). The residual cytotoxicity of L1 was measured by $^{51}$Cr-release assay, using L1210/Cr as targets.

tests. The cytotoxicity of L1 was completely absorbed by as few as $10^7$ tumour cells (Fig. 1).

The titration of L1 activity is shown in Fig. 2. The cytotoxicity was much higher in the ascitic fluid, (dilution $10^{-6}$–$10^{-7}$) than in the supernatants (dilution $10^{-3}$), of *in vitro* hybridoma cultures. Hybrids established for more than 6 months or thawed after storage at −80°C retained their capacity to secrete L1.

The cytotoxicity of L1 was completely removed by preincubation with goat anti-α mouse Ig7S (α light chains) and with goat anti-α mouse (μ-heavy chain) (Fig. 3). Since a single band has been obtained by standard immunoelectrophoresis (not reported here) and the inhibitory effects of other immunoglobulin subclasses were significatively less effective, L1 is considered to belong to the IgM subclass.

The specificity of L1 activity for L1210 leukaemia has been established by extensive screening on syngeneic and allogeneic lymphoid cells, on normal adult and foetal cells, and on a number of experimental mouse tumours.

Table II shows that spleen cells from several independent strains, both syngeneic and allogeneic, failed to remove L1 activity. The reactivity of L1 to normal and foetal tissues was studied by the indirect fluorescence method. As shown in

TABLE II.—*Absorption of L1* activity by 5 × 10⁷ spleen cells from independent strains*

| Strain | Haplotype | Absorption† |
|---|---|---|
| DBA/2 (L1210/Cr cells) | H-$2^d$ | + |
| DBA/2 (spleen cells) | H-$2^d$ | − |
| BALB/c | H-$2^d$ | − |
| $CD2F_1$ | H-$2^{d/d}$ | − |
| AKR | H-$2^k$ | − |
| C3H | H-$2^k$ | − |
| C57BL/6 | H-$2^b$ | − |
| B10.S | H-$2^s$ | − |

* The supernatant from clone E10 diluted 1:64, assayed for cytotoxicity against $^{51}$Cr-labelled L1210/Cr target cells, was the source of L1.

† Ability to completely absorb the L1 activity, assayed as in Fig. 1.

TABLE III.—*Distribution pattern of L1 antigen on DBA/2 cell surfaces*

| Cell type | Cells staining with FITC-rabbit anti-α-mouse IgG (%) — Treatment: Normal mouse serum (±s.e.) | L1 |
|---|---|---|
| L1210/Cr | 0·6±0·1 | 100±0·1 |
| L1210/Ha | 0·3±1·0 | 100±0·2 |
| Spleen | 32±6·9 | 30±5·4 |
| Marrow | 11·3±7·2 | 16·4±3·4 |
| Thymus | 1·5±0·2 | 1·3±0·1 |
| Foetal liver | 7·3±1·0 | 8·0±0·1 |
| Foetal thymus | 2·3±0·8 | 4·0±0·9 |
| | % $^{51}$Cr release by | |
| | rabbit anti-α-mouse serum | L1 |
| B-enriched population* | 78·0±0·9 | 1·0±0·1 |

* B-enriched population was obtained by treating DBA/2 spleen cells with α-Thy-1·2 antiserum and rabbit complement. Viable cells were harvested after separation on an Isopaque/Ficoll gradient. B-enriched spleen cells were labelled with $^{51}$Cr and used as targets in a complement-dependent cytotoxicity assay.

Table III, both normal and foetal tissues, including marrow, thymus, liver and spleen cells, were not stained. Moreoever, since L1210 leukaemia could have originated from a B-cell lineage (Shevach *et al.*, 1972; Cooper *et al.*, 1977), it was relevant to study its reaction with B cells. A B-enriched population, obtained by pretreatment of spleen cells from DBA/2 mouse with anti-Thy 1·2 serum and rabbit complement, was not lysed by L1 (Table III). In addition, L1 did not modify the activity of natural killer (NK) cells (Table IV).

TABLE IV.—*Effect of L1 on natural killer (NK) activity*

| Effector cells | Effectors: targets | % $^{51}$Cr-release (±s.e.) |
|---|---|---|
| BALB/c spleen cells* | 50 : 1 | 22±0·5 |
| BALB/c spleen cells + L1† | 50 : 1 | 25±1·3 |
| BALB/c spleen cells | 100 : 1 | 36±1·2 |
| BALB/c spleen cells + L1 | 100 : 1 | 34±0·9 |

* Spleen cells were incubated 30 min at 4°C with the supernatant from Clone D10 at a final dilution of 1:32. Rabbit complement was then added and cells were incubated for 45 min at 37°C. After 2 washings, cells were assayed for cytotoxicity.

† NK activity was tested in a 4 h $^{51}$Cr-release assay with $^{51}$Cr-labelled YAC-1 as target cells. Data are presented as % specific lysis, according to the formula and methods described by Cerottini (1974).

Finally, the reactivity of L1 was matched (Table V) in a complement-dependent cytotoxicity test against several lines of mouse tumours, most of them of lymphoid origin, induced by either chemical agents or oncogenic viruses. The panel of tumour cells expressed T or B cell-surface antigens: namely Thy, TL, Ly antigens, immunoglobulins, Fc and C3 receptors. Moreover, the tumour cells carried the most common structural viral antigens or non-virionic specificities that accompany oncogenic virus infection. None of the tumour lines reacted with L1. Lack of reactivity against P388 and P815 tumours rules out the possibility that the specificity recognized by L1 is a surface antigen of macrophages or mast-cells. Furthermore, a B-lymphocyte tumour line obtained by infection with Abelson virus (Abelson & Rabstein, 1970) was also completely unreactive.

Because of its high specificity and its potent cytotoxicity *in vitro*, L1 was used for immunotherapeutic studies in L1210-bearing animals. L1 and rabbit complement were inoculated, following various schedules of treatment, into leukaemic mice challenged with L1210 cells i.p. on Day 0, but failed to improve their life

TABLE V.—*Characteristics of tumour cells tested for reactivity to L1*

| Target cells | Strain of origin | Haplotype | Tumour induction | Type of tumour | % $^{51}$Cr release |
|---|---|---|---|---|---|
| L1210/Cr | DBA/2 | H-2$^{d}$ | Carcinogen | Lymphoma | +80 |
| L5178Y | DBA/2 | H-2$^{d}$ | Carcinogen | Lymphoma | 0 |
| EL4 (G+) | C57BL/6 | H-2$^{b}$ | Carcinogen | Lymphoma | −3 |
| EL4 (G−) | C57BL/6 | H-2$^{b}$ | Carcinogen | Lymphoma | −3 |
| P388 | BALB/c | H-2$^{d}$ | Carcinogen | Macrophage line | −1 |
| P815 | DBA/2 | H-2$^{d}$ | Carcinogen | Mastocytoma | 0 |
| BALB/urethan 2 | BALB/c | H-2$^{d}$ | Carcinogen | Sarcoma | −1 |
| RL ♂ 1 | BALB/c | H-2$^{d}$ | X-rays | Lymphoma | +2 |
| E ♂ G2 | C57BL/6 | H-2$^{b}$ | Gross virus | Lymphoma | −2 |
| K36 | AKR | H-2$^{k}$ | Spontaneous | Lymphoma | −1 |
| LSTRA | BALB/c | H-2$^{d}$ | Moloney virus | Lymphoma | +1 |
| YC8 | BALB/c | H-2$^{d}$ | Moloney virus | Lymphoma | +1 |
| GLV | C3H | H-2$^{k}$ | Gross virus | Lymphoma | +3 |
| Abelson | BALB/c | H-2$^{d}$ | Prednisolone + Moloney virus | B lymphosarcoma | +1 |

TABLE VI.—*Life span of L1210 leukaemic CD2F$_1$ mice treated with L1 and complement*

| L1210/Cr No. of cells on Day 0 | L1* (0·2 ml/mouse) Days and route of treatment | Rabbit complement‡ (0·4 ml/mouse) Days and route of treatment | Mean survival in days (range) |
|---|---|---|---|
| $10^3$ | — | — | 13 (12–14) |
| $10^3$ | 1, 3, 5 i.p. | — | 12 (12–13) |
| $10^3$ | — | 1, 2, 3, 4, 5 i.p. | 13 (11–15) |
| $10^3$ | 1 i.p. | 1 i.p. | 11 (9–12) |
| $10^3$ | 1, 3, 5 i.p. | 1, 2, 3, 4, 5 i.p. | 14 (14– ) |
| $10^5$ | — | — | 10 (10– ) |
| $10^5$ | 1, 3, 5 i.v. | 1, 3, 5 i.v. | 10 (9–12) |

* The ascitic fluid of pristane-primed BALB/c mice challenged with producing clones was the source of L1. The *in vitro* cytotoxicity was at $10^{-6}$ dilution.
† Checked *in vitro* for complement activity.

span over that of untreated animals (Table VI).

DISCUSSION

The L1210 lymphoblastoid cell line is a mouse lymphoma originally induced in DBA/2 mice by exposure to methylcholanthrene (Law *et al.*, 1949). As a consequence of several generations of transplants carried out in different laboratories, L1210 sublines have been originated with different degrees of immunogenicity in syngeneic hosts. The line L1210/Cr used in this study is a tumour that is weakly immunogenic in eliciting both *in vitro* immunoresponse and *in vivo* rejection. Although tumour-associated antigens in transplantable tumours have been the object of a number of studies, their intimate features are still quite undefined. The availability of monoclonal antibodies might facilitate the biochemical and functional characterization of TAA. In the present report we describe an IgM monoclonal antibody (L1) strictly specific for an antigen associated with L1210 leukaemia. The antibody (L1) obtained from ascitic fluid has shown high cytotoxicity at dilutions as high as $10^{-6}$–$10^{-7}$. The L1 antigenic specificity was expressed by every cell in the L1210 population, all of which stained in the indirect fluorescence assay. The L1210 cells cultured *in vitro* showed decreased susceptibility to L1 cytotoxicity, probably because of increased shedding of the antigen into the supernatant (unpublished observations from this laboratory).

Extensive screening with normal and malignant cells ruled out any reactivity of L1 to histocompatibility antigens, to antigens defining different subsets of lymphoid cells and to surface determinants of macrophages, mast cells, or marrow cells. Moreover, L1 did not recognize foetal tissues, namely liver, thymus and spleen, with which L1210 leukaemia might share some differentiation antigens. Finally, a panel of laboratory tumour cells, transformed by different mechanisms and expressing on their surfaces a number of antigenic determinants and receptors, did not express the antigen recognized by L1. The specificity of L1 to L1210 leukaemia cells was established by its lack of reactivity with a variety of target cells and from the schedule of hybridization (syngeneic immunization and mouse–mouse fusion). In spite of the high *in vitro* cytotoxicity of L1 and lack of toxicity to the host tissues, attempts at immunotherapy of L1210 mice were unsuccessful. Other schedules of treatment, not reported here, were also ineffective. Complement inactivation *in vivo*, neutralization of L1 by antigens circulating in the blood or present in the ascitic fluid are first among the hypotheses that should be experimentally tested in attempts to explain the negative *in vivo* findings. Antigenic modulation by L1 itself, or poor binding to target tumour cells, might also be worth attention. Whatever the reason for the failure of these and other (Houston *et al.*, 1980; Ritz *et al.*, 1980) preliminary attempts at serological therapy, the exploitation of the monoclonal antibody technology in experimental cancer might be furthered by studies with L1. Since L1210 leukaemia has been widely used over the last 30 years and there is good knowledge of its *in vivo* and *in vitro* biological properties, studies with this experimental model might easily and quickly provide some essential pieces of information. The high specificity of L1, and its lack of reactivity to normal tissues, might make it an optimal tool in carrying toxic agents to target cells or developing experimental methodologies for highly sensitive immunodiagnosis.

This research was supported in part by PFCCN from C.N.R., Rome, Italy.

We wish to thank Dr Giorgio Corte, Istituto di Biochimica, Genoa, Italy, for the generous gift of P3X63/Ag8 myeloma cells.

### REFERENCES

ABELSON, H. T. & RABSTEIN, L. S. (1970) Lymphosarcoma: Virus-induced thymic-independent disease in mice. *Cancer Res.*, **30**, 2213.

BLACK, P. H. (1980) Shedding from normal and cancer-cell surfaces. *N. Engl J. Med.*, **303**, 1415.

BONMASSAR, E., BONMASSAR, A., VADLAMUDI, S. & GOLDIN, A. (1970) Immunological alteration of leukemic cells *in vivo* after treatment with an antitumor drug. *Proc. Natl Acad. Sci.*, **66**, 1089.

BOYSE, E. A., STOCKERT, E. & OLD, L. J. (1967) Modification of the antigenic structure of the cell membrane by thymus leukemia (TL) antibody. *Proc. Natl Acad. Sci.*, **58**, 954.

CEROTTINI, J. C., ENGERS, H. D., MACDONALD, H. R. & BRUNNER, K. T. (1974) Generation of cytotoxic T-lymphocytes *in vitro*: Response of normal and immune spleen cells in mixed lymphocyte cultures. *J. Exp. Med.*, **140**, 703.

COOPER, S. M., SAMBRAY, Y. & FRION, G. (1977) Isolation of separate Fc receptors for IgG complexed to antigen and native IgG from a murine leukemia. *Nature*, **270**, 253.

GUNN, B., EMBLETON, M. J., MIDDLE, J. G. & BALDWIN, R. W. (1980) Monoclonal antibody against a naturally occurring rat mammary carcinoma. *Int. J. Cancer*, **26**, 325.

HAWRYLKO, E. (1978) Mechanisms by which tumors escape immune destruction In *Handbook of Cancer Immunology, Vol. 2,* (Ed. Waters). New York: Gareand STPM Press. p. 1.

HEWITT, H. B., BLAKE, E. R. & WALDER, A. S. (1976) A critique of the evidence for active host defence against cancer, based on personal studies of 27 murine tumors of spontaneous origin. *Br. J. Cancer*, **33**, 241.

HOUSTON, L. L., NOWINSKI, R. C. & BERNSTEIN, I. D. (1980) Specific *in vivo* localization of monoclonal antibodies directed against the Thy 1.1 antigen. *J. Immunol.*, **125**, 837.

KITANO, M., MIHICH, E. & PRESSMAN, D. (1972) Antigenic differences between leukemia L1210 and a subline resistant to methylglyoxal-bis(guanylhydrazone). *Cancer Res.*, **32**, 181.

KLEIN, G., SJÖGREN, H. O., KLEIN, E. & HELLSTRÖM, K. E. (1960) Demonstration of resistance against methylcholanthrene induced sarcomas in primary autochthonous hosts. *Cancer Res.*, **20**, 1561.

LAW, L. W., DUNN, T. B., BOYLE, P. J. & MILLER, J. M. (1949) Observations on the effect of a folic acid antagonist on transplantable lymphoid leukemias in mice. *J. Natl Cancer Inst.*, **10**, 179.

LITTLEFIELD, J. W. (1964) Selection of hybrids from matings of fibroblasts *in vitro* and their presumed recombinants. *Science*, **145**, 709.

MIHICH, E. & KITANO, M. (1971) Differences in the immunogenicity of leukemia L1210 sublines in DBA/2 mice. *Cancer Res.*, **31**, 1999.

MOTTA, R. (1971) Passive immunotherapy of leukemia and other cancers. *Adv. Cancer Res.*, **14**, 161.

NADLER, L. M., STASHENKO, P., HARDY, R. & SCHLOSSMAN, S. F. (1980*a*) A monoclonal antibody defining a lymphoma-associated antigen in man. *J. Immunol.*, **125**, 570.

NADLER, L. M., STASHENKO, P., HARDY, R. & 5 OTHERS (1980*b*) Serotherapy of a patient with monoclonal antibody directed against a human lymphoma-associated antigen. *Cancer Res.*, **40**, 3147.

NICOLIN, A., BINI, A., CORONETTI, E. & GOLDIN, A. (1974) Cellular immune response to a drug-treated L5178Y lymphoma subline. *Nature*, **251**, 654.

NICOLIN, A., VERONESE, F., MARELLI, O. & GOLDIN, A. (1980) Immunological resistance to L1210 leukemia induced by viable L1210/DTIC cells. *Cancer Immunol. Immunother.* **9**, 43.

NOWINSKI, R. C., LOSTROM, M. E., TAM, M. R., STONE. M. R. & BURNETTE, W. N. (1979) The isolation of hybrid cell lines producing monoclonal antibodies against the p15(E) protein of ecotropic murine leukemia viruses. *Virology*, **93**, 111.

OLD, L. J. & BOYSE, E. A. (1964) Immunology of experimental tumors. *Ann. Rev. Med.*, **15**, 167.

OZATO, K., MAYER, N. & SACHS, D. H. (1980) Hybridoma cell lines secreting monoclonal antibodies to mouse H-2 and Ia antigens. *J. Immunol.*, **124**, 533.

PREHN, R. T. & MAIN, J. M. (1957) Immunity to methylcholanthrene induced sarcomas. *J. Natl Cancer Inst.*, **18**, 769.

RITZ, J., PESANDO, J. M., MCCONARTY, J. N., LAZARUS, H. & SCHLOSSMAN, S. F. (1980) A monoclonal antibody to human acute lymphoblastic leukaemia antigen. *Nature*, **283**, 583.

ROSENBERG, S. A. & TERRY, W. D. (1977) Passive immunotherapy of cancer in animals and man. *Adv. Cancer Res.*, **25**, 323.

SHEVACH, E. M., STOBO, J. D. & GREEN, I. (1972) Immunoglobulin and teta-bearing murine leukemias and lymphomas. *J. Immunol.*, **108**, 1146.

SIMRELL, C. R. & KLEIN, R. A. (1979) Antibody response of tumor bearing mice to their own tumors captured and perpetuated as hybridomas. *J. Immunol.*, **123**, 2286.

STACKPOLE, C. W., CREMONA, P., LEONARD, C. & STREMMEL, P. (1980) Antigenic modulation as mechanism for tumor escape from immune destruction: Identification of modulation-positive and modulation-negative mouse lymphomas with xenoantisera to murine leukemia virus gp 70. *J. Immunol.*, **125**, 1715.